# A systematic review of the Woven EndoBridge device—do findings in pre-clinical animal models compare to clinical results?

**DOI:** 10.1007/s00701-023-05638-y

**Published:** 2023-06-08

**Authors:** René Aquarius, Danique Elbertsen, Joost de Vries, Hieronymus D. Boogaarts, Kimberley E. Wever

**Affiliations:** 1 Department of Neurosurgery, Radboud University Medical Center, Geert Grooteplein Zuid 10, 6525GA Nijmegen, Gelderland The Netherlands; 2 Department of Anesthesiology, Pain and Palliative Medicine, Radboud University Medical Center, Nijmegen, Gelderland The Netherlands

**Keywords:** Endovascular procedures, Intracranial aneurysm, Woven endobridge device, Systematic review

## Abstract

**Background:**

The Woven Endobridge (WEB) is designed to treat intracranial wide-neck bifurcation aneurysms, preventing subarachnoid hemorrhage. The translational value of animal models used for WEB device testing is unknown. With this systematic review, we aim to identify the existing animal models used in testing the WEB device and compare the efficacy and safety outcomes to those of prospective clinical studies.

**Methods:**

This study was funded by ZonMw: project number 114024133. A comprehensive search was performed in PubMed and in EMBASE via the Ovid interface. The following exclusion criteria were used: 1) not an original full-length research paper, 2) not an in vivo animal study or a human study, 3) no WEB implantation, 4) if in humans: not a prospective study. The SYRCLE risk of bias tool (animal studies) and the Newcastle–Ottawa quality assessment scale for cohort studies (clinical studies) were used to assess risks of bias. A narrative synthesis was performed.

**Results:**

Six animal studies and 17 clinical studies met the inclusion criteria. The rabbit elastase aneurysm model was the only animal model used to assess WEB device performance. Safety outcomes were never reported in animal studies. Efficacy outcomes were more heterogeneous in animal studies than in clinical studies, which could be due to limited external validity of the animal models in terms of aneurysm induction and dimensions. Both animal and clinical studies were predominantly single-arm studies, and were at unclear risk of several types of bias.

**Conclusions:**

The rabbit elastase aneurysm model was the only pre-clinical animal model used to assess WEB device performance. Safety outcomes were not evaluated in animal studies and could therefore not be compared to clinical outcomes. Efficacy outcomes were more heterogeneous in animal studies than in clinical studies. Future research should focus on improving methodology and reporting in order to draw accurate conclusions on the performance of the WEB device.

**Supplementary Information:**

The online version contains supplementary material available at 10.1007/s00701-023-05638-y.

## Introduction

The Woven Endobridge (WEB) device has been designed to occlude wide-neck bifurcation aneurysms (WNBAs), a type of intracranial aneurysm that is notoriously difficult to treat [4, 15]. The WEB device is a self-expanding mesh that, when placed inside the IA through an endovascular procedure, will disrupt blood flow entering the aneurysm and help promote thrombosis. The goal is to exclude the WNBA from the circulation, thereby preventing subarachnoid hemorrhage in unruptured cases, and recurrent hemorrhage in those who present with rupture.

New endovascular devices generally undergo some degree of pre-clinical testing before implementation in the clinic. However, the animal models for aneurysm repair used may vary in terms of the study design, the method used for model induction, the dimensions of the aneurysms produced and the outcomes measured, all of which have different implications for their internal and external validity. Moreover, the models’ translational value may differ depending on the type of device tested. With this systematic review we aim to identify the existing animal models used in testing the WEB device and compare the efficacy and safety outcomes of these models to the efficacy and safety outcomes of prospective clinical data.

## Methods and materials

### Reporting and protocol

This review is reported according to the PRISMA guidelines. The review methodology was recorded a priori in accordance with SYRCLE’s systematic review protocol for animal intervention studies [7] and registered in the international prospective register of systematic reviews PROSPERO (protocol ID: CRD42021252964). No amendments to the review protocol were made during the study.

### Search strategy

A comprehensive search was performed in PubMed and in EMBASE via the Ovid interface. The full comprehensive search strategy is presented in Online Resource 1. Our aim was to identify all studies performed in animals or humans on the efficacy or safety of the WEB device in aneurysm repair. The full search strategy was based on the components “Woven Endobridge device,” “aneurysm,” and “intrasaccular implant.” The search was performed on June 6th 2021, without applying any language restrictions, date restrictions or search filters.

### Study selection

After removal of duplicates using Endnote (X9.2, Clarivate Analytics, United States), the search results were imported into the online screening platform Rayyan (Rayyan.ai, Rayyan Systems Inc., United States). In the first phase, references were screened for eligibility by two reviewers (RA and DE) based on title and abstract. Eligible papers were subsequently screened for final inclusion (by RA and DE) based on the full text. In both phases, studies were excluded if at least one of the following exclusion criteria was applicable:

1. Not an original full-length research paper (no letters, reviews, etc.)
2. Not an in vivo animal study or a human study
3. No Woven Endobridge device implantation
4. If in humans: not a prospective study

If a full text reference could not be found online or at the Radboudumc library, the corresponding author was contacted through email with a request for a full text version of the manuscript. A reminder was sent to all authors who did not respond within two weeks. If authors did not respond to this reminder within 2 weeks, the reference was excluded.

In both phases, the two reviewers independently assessed each reference and were blinded to each other's assessment. In case of discrepancies, reviewers first attempted to reach agreement through discussion. If no consensus could be achieved, the opinion of a third reviewer (KW) would be decisive.

### Data extraction

Bibliographic details including author, journal and year of publication were extracted from each included publication.

For animal studies, the following characteristics were extracted: species, strain, sex, weight and/or age, method of aneurysm induction, location of aneurysm, type of aneurysm, size of aneurysm, aneurysm patency after induction, the way aneurysm occlusion was determined after treatment, WEB device sizing and timing of WEB device placement (at the time of aneurysm creation or n days after aneurysm creation), aneurysm occlusion (percentage, Raymond Roy scale, etc.), all-cause mortality (incidence), complications (incidence; overall and per type).

For prospective human studies, the following characteristics were extracted: sex, weight and/or age, co-morbidities, location of intracranial aneurysm, type of aneurysm, size of aneurysm, the way aneurysm occlusion was determined after treatment, WEB device sizing, aneurysm occlusion (percentage, Raymond Roy scale, etc.), all-cause mortality (incidence), complications (incidence; overall and per type), functional outcome (mRS, Glasgow coma scale, etc.).

All data was extracted by one author (DE) and checked for completeness by a second author (RA) independently. Discrepancies were discussed between both authors until agreement was reached. When data were missing, the corresponding author was contacted through email with a request for the raw data file. A reminder was sent to all authors who did not respond within two weeks. If authors did not respond to this reminder within two weeks, data were excluded from the study.

### Outcome measures and data synthesis

From the extracted data, aneurysm remnant (%) was calculated from the number of patients available at the time of follow-up. The aneurysm remnant per point in time was graphically presented for both pre-clinical and clinical studies. Differences in aneurysm remnants between pre-clinical and clinical studies were described. Complications described in animals and humans were listed in a table and were compared to identify similarities.

No meta-analysis was planned as we expected that most of the relevant literature would not have a control group included.

### Risk of bias assessment

Two authors (RA and DE) independently assessed the risk of bias for each included study, blinded to each other's assessment. Both reviewers resolved discrepancies through discussion. If no consensus could be achieved, the opinion of a third author (KW) would be leading.

Animal studies were assessed using SYRCLE’s risk of bias tool [13] with the addition of the following 5 questions, which served as study quality indicators [16, 34]:

1. Was any randomization reported at any level of the experiment? (Y/N).
2. Was any blinding reported at any level of the experiment? (Y/N).
3. Was a power or sample-size calculation reported? (Y/N).
4. Was a conflict of interest statement reported? (Y/N).
5. Was a prespecified / preregistered protocol reported? (Y/N).

For studies without a second arm, items 1 to 6 of SYRCLE’s risk of bias tool were not applicable and therefore omitted from the assessment.

Clinical studies were assessed using the Newcastle–Ottawa quality assessment scale for cohort studies. In case single-arm studies were included, all questions in the comparability category and the question regarding selection of the non-exposed cohort in the selection category would be omitted. Thus, for single-arm studies, the maximal achievable score was 7, rather than 9 stars.

## Results

### Inclusions

**Fig. 1:**
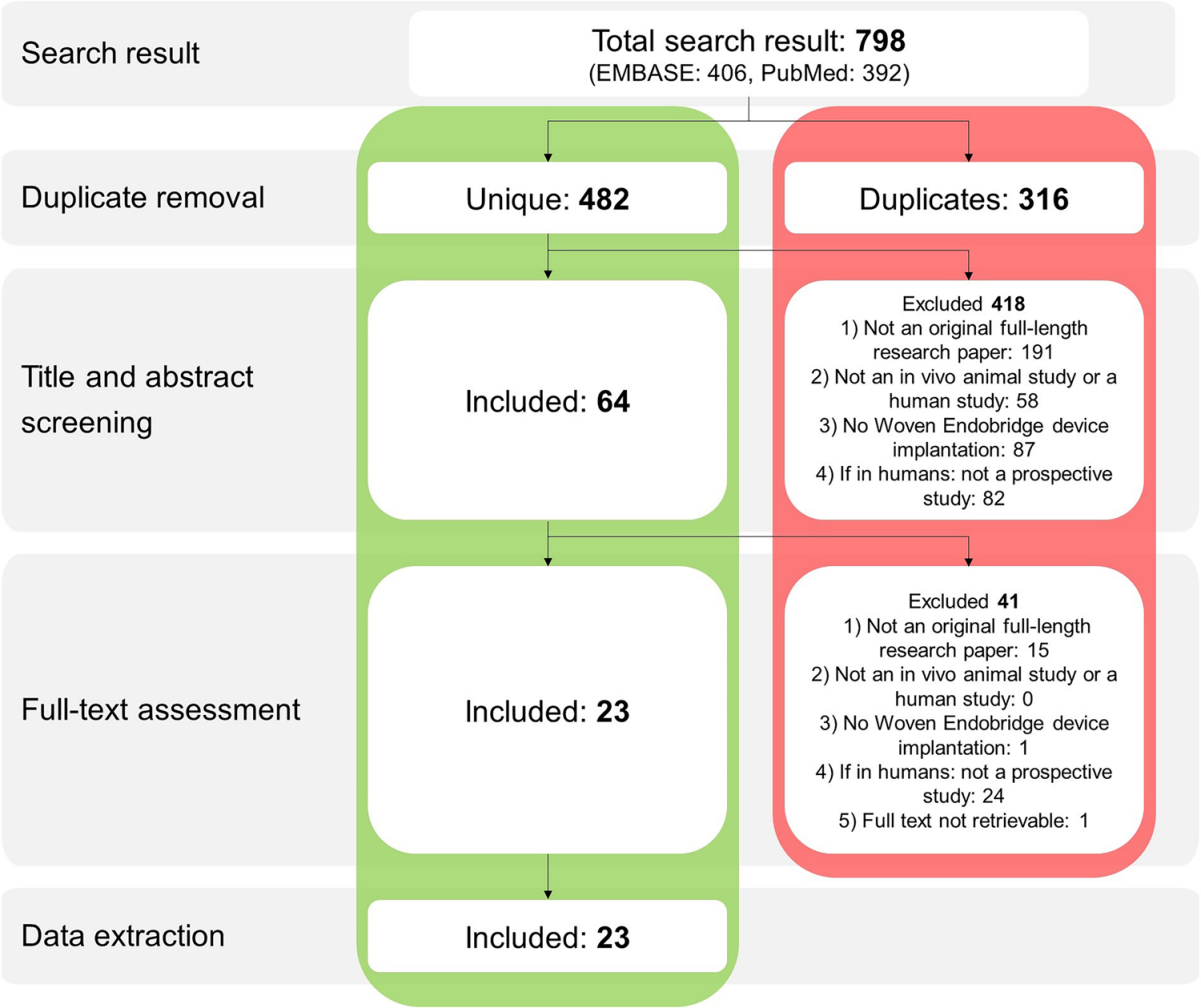
Flow chart depicting the study inclusion process

A total of 23 studies were included for data extraction (Fig. 1). A full reference list of all included studies is presented in Online Resource 2. No authors were contacted as all full text publications were all found online or through our institute’s library.

### Study characteristics

**Fig. 2:**
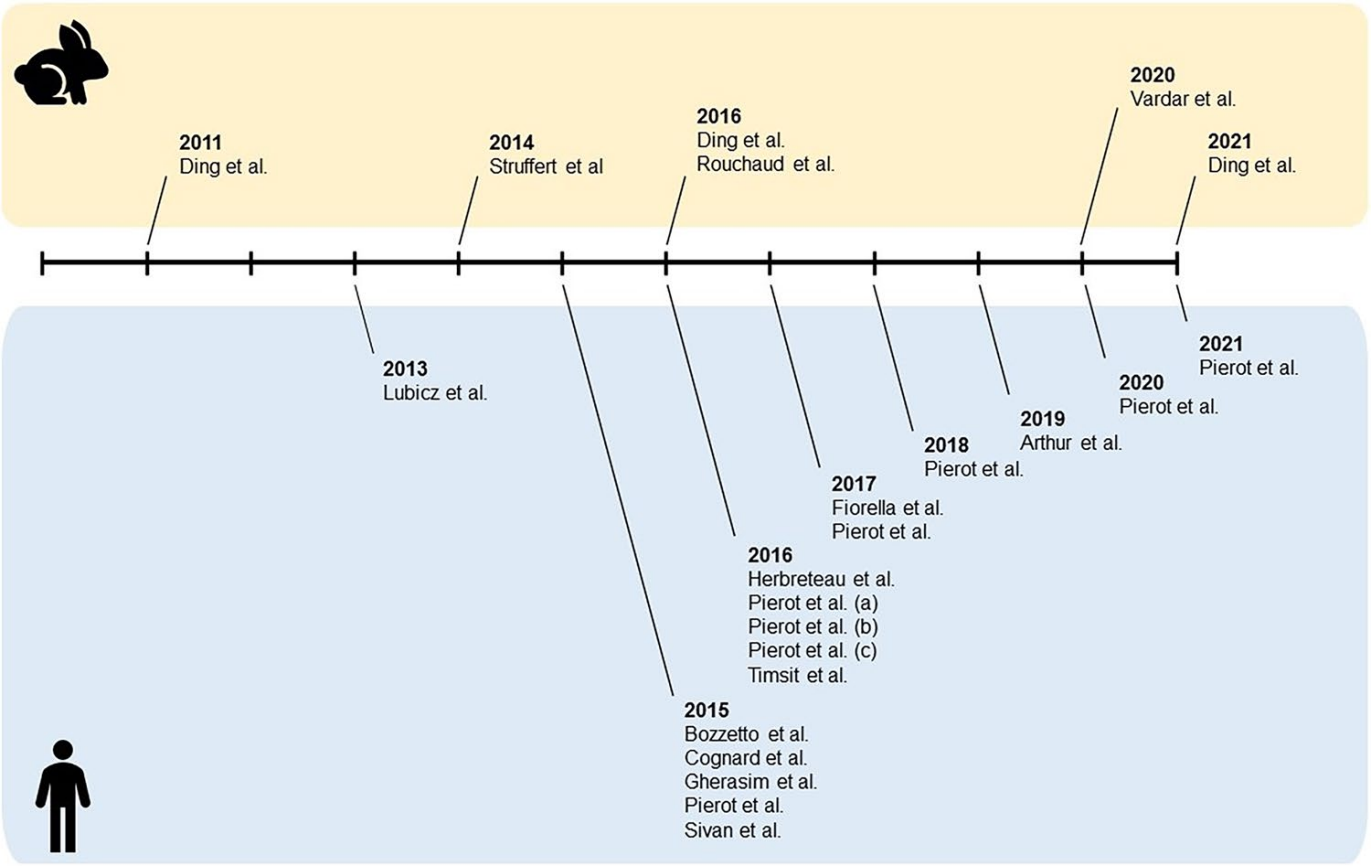
Timeline depicting when the pre-clinical animal studies (upper half) and prospective clinical studies (lower half) were published. Bibliographical details of these studies can be found in Online Resource 2

The 23 included studies were comprised of 6 pre-clinical animal studies published between 2011 and 2021 and 17 prospective clinical studies published between 2013 and 2021 (Fig. 2).

### Animal study characteristics

All 6 pre-clinical animal studies included in this systematic review utilized the rabbit elastase model, in which a segment of the right common carotid artery is ligated and treated with elastase in order to induce the aneurysm over time. In these 6 studies, a total of 205 New Zealand white rabbits with 205 elastase-induced aneurysms were used and were implanted with a WEB device at least 3 weeks after aneurysm induction.

Sex and weight of the animals was reported in 1 study (male, weight range of 3.8–4.2 kg). None of the studies reported the age of the animals. Aneurysm dimensions were reported in 2 studies (60 animals). Aneurysm width ranged from 3.5 to 3.9 mm, aneurysm neck size ranged from 2.7 to 3.4 mm. The type of WEB device used was specified in every included study, details on the sizing of the WEB devices were specified in 2 studies. None of the studies used a control group in which the aneurysm was treated conservatively or with a different implant. All characteristics of the animal studies are listed in Online Resource 3.

### Clinical study characteristics

Of the 17 prospective clinical studies, 8 studies described an original patient cohort. The remaining 9 studies described additional follow-up of previously defined patient cohorts. The 8 studies with original cohorts comprised a total of 365 patients with 367 IAs located in the middle cerebral artery (*n* = 152 (41.4%)), anterior communicating artery (*n* = 97 (26.4%)), basilar artery tip (*n* = 93 (25.3%), internal carotid artery tip (*n* = 24 (6.5%)) and the vertebral artery—posterior inferior cerebellar artery junction (*n* = 1 (0.3%)).

The 8 studies with original cohorts all specified the sex of the included patients: 247 female (67.7%) and 118 male (32.3%). Mean age was reported in all 8 studies with original cohorts and ranged from 54.2 to 59.3 years. A statement regarding aneurysm dimensions was made in each of the 8 included studies with original cohorts. Mean aneurysm neck width ranged from 4.6 to 6.5 mm, based on 7 of these 8 studies. Mean aneurysm dome width ranged from 6.2 to 8.1 mm, based on 5 of these 8 studies. The remaining studies used either an unclear dimension: mean aneurysm diameter (8.2 mm, *n* = 1), mean aneurysm size (6.7 mm, *n* = 1) or cut-off values to describe the aneurysm dimensions (52/62 aneurysms (83.9%) with a maximum aneurysm width of < 10 mm and 57/62 aneurysms (91.3%) with an aneurysm neck width of ≥ 4 mm, *n* = 1). WEB sizing was reported in 7 out of 8 studies with an original cohort. However, the detail in reporting ranged from a general statement (4 studies) to a detailed description of the sizes used (2 studies). One study detailed the sizes of the most frequently used WEB devices, but no statement was made regarding the remaining WEB devices. None of the included studies used a control group or a group in which the aneurysm was treated conservatively or with a different implant. All characteristics of the human studies are presented in Online Resource 4.

### Risk of bias assessment—animal studies

**Fig. 3:**
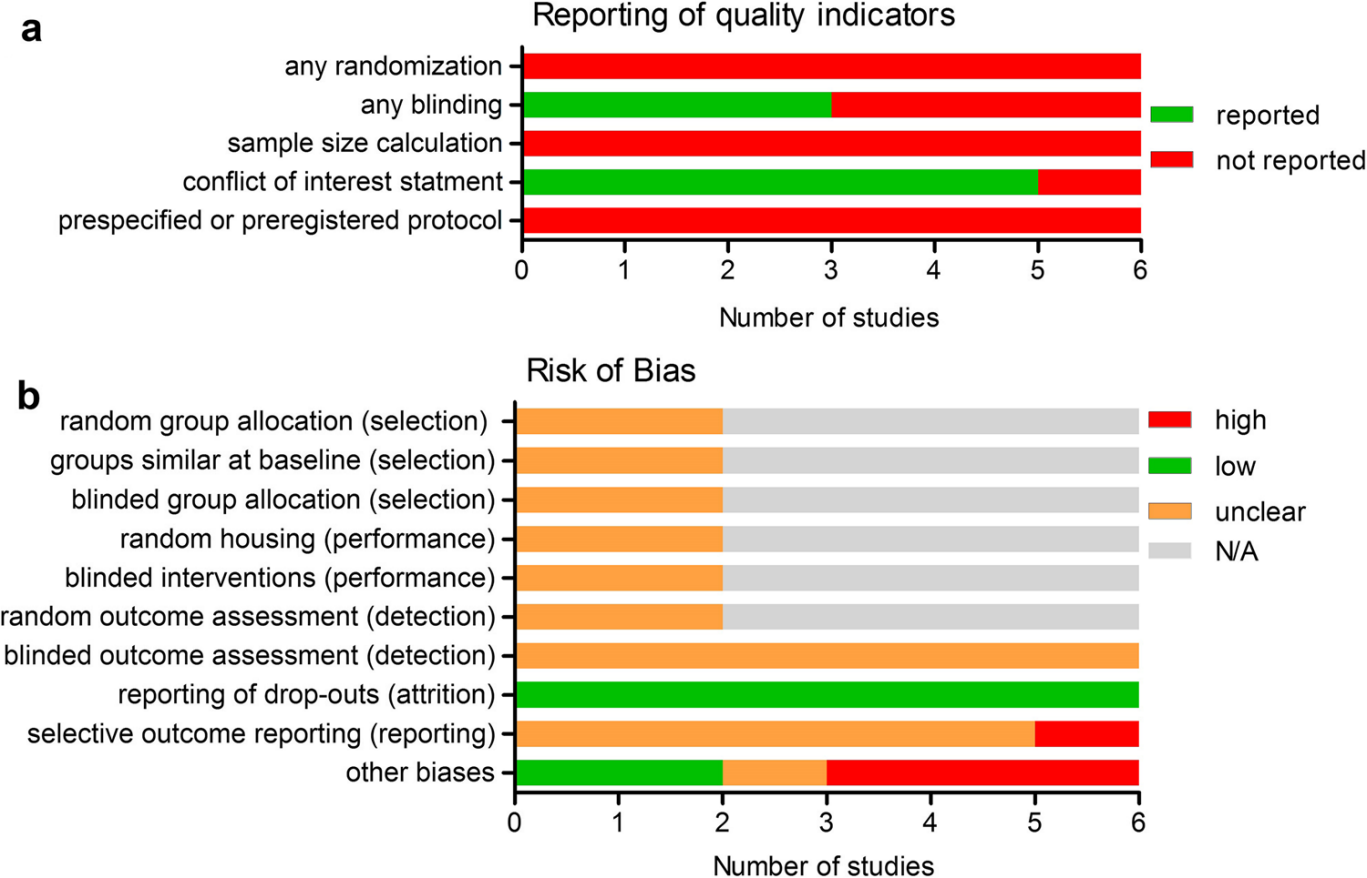
Reporting of quality indicators (**A**) and risk of bias assessment (**B**) for the included animal studies. Bibliographical details of these studies can be found in Online Resource 2

In none of the included animal studies any statement was given regarding randomization, sample size calculation or the existence of a preregistered study protocol. In 3 out of 6 studies (50%) blinding was mentioned and in 5 out of 6 studies (83.3%) a conflict of interest statement was reported (Fig. 3).

Animal studies clearly reported that all animals entering the study were eventually analyzed, resulting in a low risk of attrition bias. For “other biases,” we assessed potential conflicts of interest statements, which resulted in a low risk of bias for 2 studies (no conflict of interest) and a high risk of bias in 3 studies (a reported conflict of interest). One study had a high risk of selective outcome reporting, due to a focus on a subgroup analysis that was not mentioned in the methods section. Assessment of items 1 to 6 of SYRCLE’s risk of bias tool were omitted for 4 studies as these were single arm studies (Fig. 3).

### Risk of bias assessment—clinical studies

The Newcastle–Ottawa scores ranged from 4 to 6 stars (Table 1). None of the included clinical studies had a control group, therefore the “comparability” category of the Newcastle–Ottawa scale could not be assessed and none of the studies received the maximum score of 9 stars. Furthermore, study cohorts sometimes misrepresented the target patient population, as not all included aneurysms were WNBAs. In addition, outcome assessment was sometimes not performed by independent researchers. Finally, patients were often lost during follow-up, especially for later time points, with numbers of missing patients between 20 and 40% of the initial cohort. Although some patients had died from unrelated causes, in many cases it was unclear why these patients were lost to follow-up.

**Table 1:** The Newcastle–Ottawa quality assessment scores for all included studies. Note that the comparability category was not applicable as there were no studies included with a control group. Within the selection category the item selection of the non-exposed cohort was not applicable, as there were no studies with a non-exposed cohort

Study	Selection (max. 4 stars)	Comparability (max. 2 stars)	Outcome (max. 3 stars)	Final Rating (max. 9 stars)
Arthur et al. [2]	⋆⋆⋆	N/A	⋆⋆⋆	⋆⋆⋆⋆⋆⋆
Bozzetto et al. [3]	⋆⋆⋆	N/A	⋆	⋆⋆⋆⋆
Cognard et al. [5]	⋆⋆⋆	N/A	⋆⋆	⋆⋆⋆⋆⋆
Fiorella et al. [9]	⋆⋆⋆	N/A	⋆⋆⋆	⋆⋆⋆⋆⋆⋆
Gherasim et al. [14]	⋆⋆⋆	N/A	⋆	⋆⋆⋆⋆
Herbreteau et al. [12]	⋆⋆⋆	N/A	⋆⋆	⋆⋆⋆⋆⋆
Lubicz et al. [18]	⋆⋆⋆	N/A	⋆^a^	⋆⋆⋆⋆
Pierot et al. [23]	⋆⋆⋆	N/A	⋆⋆	⋆⋆⋆⋆⋆
Pierot et al. [24]	⋆⋆⋆	N/A	⋆⋆⋆	⋆⋆⋆⋆⋆⋆
Pierot et al. [25]	⋆⋆⋆	N/A	⋆⋆⋆	⋆⋆⋆⋆⋆⋆
Pierot et al. [30]	⋆⋆⋆	N/A	⋆⋆	⋆⋆⋆⋆⋆
Pierot et al. [29]	⋆⋆⋆	N/A	⋆⋆⋆	⋆⋆⋆⋆⋆⋆
Pierot et al. [26]	⋆⋆⋆	N/A	⋆⋆	⋆⋆⋆⋆⋆
Pierot et al. [28]	⋆⋆⋆	N/A	⋆⋆⋆	⋆⋆⋆⋆⋆⋆
Pierot et al. [31]	⋆⋆⋆	N/A	⋆⋆	⋆⋆⋆⋆⋆
Sivan et al. [33]	⋆⋆⋆	N/A	⋆⋆	⋆⋆⋆⋆⋆
Timsit et al. [35]	⋆⋆	N/A	⋆⋆	⋆⋆⋆⋆

^a ^Scores differed between clinical outcome and angiographic outcome, but both resulted in thesame number of stars in the outcome category.

### Data synthesis—timing

The included animal experiments were published between 2011 and 2021 and the prospective clinical studies were published between 2013 and 2021 (Fig. 2).

### Data synthesis—reporting

Key animal model and aneurysm characteristics were often not reported in the included animal studies, limiting their transparency and reproducibility (Online Resource 3). Key patient and aneurysm characteristics were described in every clinical study included (Online Resource 4).

Key device characteristics, such as WEB device type (dual layer, single layer or single layer sphere) was documented in every included animal study (100%), while 4 animal studies did not report on WEB device sizes used (67%) (Online Resource 3). Type of WEB device used was documented in all but one of the included clinical studies (94%). Three animal studies did not report on WEB device sizes used (18%) (Online Resource 3).

All included animal studies reported on WEB device efficacy (100%), while none of these studies reported on the safety associated with WEB device implantation (0%). It is unclear if safety was assessed and not reported or if it was not assessed at all (Online Resource 3). This in in stark contrast with the clinical studies, in which 15 out of 17 reported on device efficacy (88%) and 15 out of 17 reported on safety (88%) after WEB implantation (Online Resource 3).

### Data synthesis—methodology

All included animal studies used New Zealand white rabbits with an elastase-induced aneurysm in an extracranial vessel (the right common carotid artery). No other aneurysm location, aneurysm type or species was used for assessment of the WEB device. The average width of aneurysms in the included animal studies (range 3.5–3.9 mm) was smaller than that of aneurysms reported in human studies (range 6.2–8.1 mm). The average aneurysm neck size was also smaller in the animal studies (range 2.7–3.4 mm) than in the human studies (range 4.6–6.5 mm) (Online Resources 3 and 4).

The performance of the WEB device was not compared to a (negative) control group in any of the 23 included studies (0%) (Online Resources 3 and 4).

Clinical cohorts were often re-used in different publications, sometimes to report on additional follow-up data when it was available. However, in some cases cohorts were merged or parts of previously described cohorts were re-used to present additional follow-up data. This caused a lack of transparency as it is often unclear which data were reused and why they were reused (marked orange in Online Resource 4).

### Data synthesis—efficacy results

**Fig. 4:**
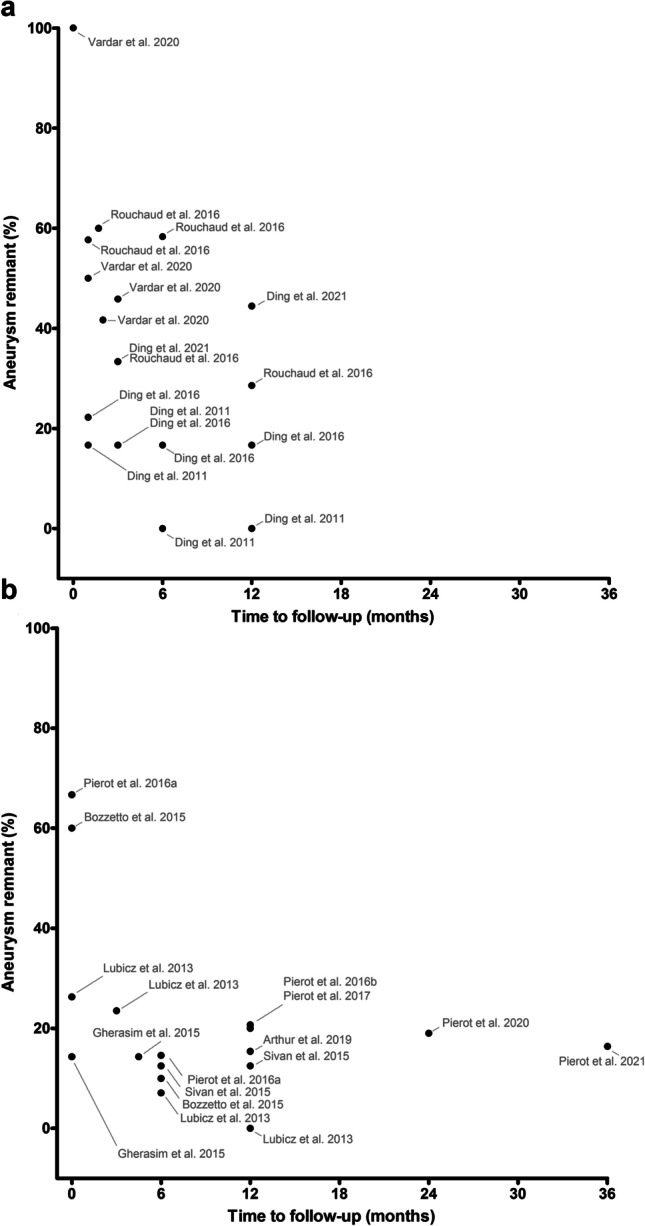
The percentage of aneurysm remnants per timepoint for the included pre-clinical animal (**A**) and prospective clinical studies (**B**). Bibliographical details of all mentioned studies can be found in Online Resource 2

In general, aneurysm remnant percentage tended to be higher for animal studies than for clinical studies. Directly after WEB device implantation, 1 animal study showed that all aneurysms had remnants, while in the clinical studies the percentage of aneurysm remnant ranged from 14.3 to 66.7%. In the first year of follow-up after WEB implantation, aneurysm remnants varied from 0 to 44.4% in the animal studies, while it ranged from 0 to 23.5% in the human studies. Follow-up beyond 1 year was only available for prospective clinical studies (Fig. 4).

### Data synthesis—safety results

None of the included animal studies reported on WEB device safety. Therefore, a comparison of safety data between pre-clinical and clinical data was not possible (Online Resources 3 and 4).

Safety data of the included clinical studies demonstrated similar safety issues, mainly related to thromboembolic events or intraoperative aneurysm rupture. Five out of 17 studies (29%) published safety data which had previously been published. This led to a discrepancy between two studies which presented data from the same cohort at the same moment in time: one study reported 9 thromboembolic events and the other 10 (See Pierot et al. 2015 and Pierot et al. 2016b in Online Resource 4).

## Discussion

The goal of our systematic review was to identify the existing animal models used in testing the WEB device and compare the efficacy and safety outcomes of these models to the efficacy and safety outcomes of prospective clinical data.

### Animal model

Although the rabbit elastase model offers hemodynamic and morphologic similarity to humans and likely mimics the tissue response [6], it also has limitations regarding its external validity. First, aneurysms in the rabbit elastase model are too small to be labeled as wide-necked and are not bifurcation aneurysms and therefore do represent WNBAs. Another downside of this model is that the aneurysm wall in the rabbit elastase model is thick and homogenous and does not show the same signs of degeneration as seen in unstable human aneurysms [10]. Therefore, issues like aneurysm wall perforation during the procedure are unlikely to happen in this animal model, in contrast to clinical practice. In general, it is important to use different animal models to reflect different aspects of the simulated pathology [32] to make sure that results of device testing are generalizable to a broader context. Many different animal models have previously been described to simulate intracranial aneurysms [10, 13, 22, 37] and researchers within the field are encouraged to use more than one animal model when testing endovascular implants.

### Safety

It is unclear whether no safety endpoints were reached and simply not reported or whether safety outcomes were not assessed. Complications in the rabbit elastase model are extremely difficult to notice when not explicitly assessing safety outcomes. Rabbits are prey animals and instinctively hide handicaps to avoid predation [20]. Consequences of a complication, such as a thromboembolic event, are likely to go unnoticed as the extracranial aneurysm probably will lead to less life threatening situations than when they would occur intracranially. Powering an animal study to detect safety issues is very difficult: e.g., a pre-clinical study to detect a rare complication (0% in control group vs. 2% in intervention group, 1-β of 0.8 and α of 0.05) would require 387 animals in each group. However, registering complications and deaths during pre-clinical studies can be valuable, even when the study is powered for device efficacy. For example, in a pre-clinical animal study on flow diverter efficacy, the authors mentioned encountered complications and deaths of animals in every group, including flow diverter migration leading to an incompletely sealed aneurysm and subsequent rupture [1]. This observation could warn clinicians and researchers that this type of complication is possible when using flow diverters. Another strategy could be to perform an autopsy at the end of a pre-clinical animal study to uncover hidden issues such as thromboembolic events and aid in a more rigorous assessment of safety in general after WEB implantation.

### Efficacy

Aneurysm remnant percentages in animal studies were higher than those found in the prospective clinical studies. This is puzzling as the rabbit elastase model produces side-wall aneurysms with a narrow neck, which should be more favorable for swift occlusion than the WNBAs encountered in the human studies [27]. Four possible explanations can be given for this unexpected discrepancy:

1. The rabbit elastase model is not an accurate representation of the clinical situation and results generated using this model can only be extrapolated to some extent;
2. Low numbers of animals used in pre-clinical experiments might lead to distorted results of aneurysm occlusion data after WEB device implantation;
3. Clinical trial results are at risk of attrition bias due to patients who are lost to follow-up, potentially causing patients with aneurysm remnants to be unavailable for further analysis;
4. Selection bias might occur in clinical studies, resulting in a selection of patients with aneurysms that are somehow favorable for occlusion after WEB treatment.

It is difficult to pinpoint the exact reason(s) for the discrepancies found in the efficacy in animal and clinical studies. However, using different animal models with sufficiently sized groups will help to facilitate improvement. Moreover, investigators of clinical studies should implement and rapport on measures to minimize attrition and selection bias.

### Risk of bias

Poor reporting in animal studies is associated with poor experimental design and can introduce bias and exaggerated effect sizes [19, 37]. The incomplete reporting was reflected in the risk of bias assessment: many items could not be assessed (unclear risk of bias) or were flagged as high risk of bias. This could be significantly improved if authors adhered to the Animals in Research: Reporting In Vivo Experiments (ARRIVE) guidelines [22] and if journals and peers enforced these guidelines to improve the quality of animal studies [17].

The reporting of clinical studies was of higher quality than the animal studies. However, all included studies lack a control group, which makes it difficult to interpret efficacy and safety as no comparison to conservative treatment or treatment with other endovascular devices can be made. The difficulty with comparing single-arm WEB device studies to previously published studies on other devices is that key aspects often differ, thereby hampering direct comparison. For example, differences in aneurysm size, location and rupture status, differences in outcomes measured and methodological differences in study design can all impact comparability between studies.

In 9 out of the 17 included prospective clinical studies (53%), a previously used patient cohort was used to conduct the study. Studies sometimes revisited previously described cohorts to report on long-term results, but sometimes (parts of) patient cohorts were mixed, resulting in an unclear overview of the available data. Data were often published more than once and this was not always transparently described (Online Resources 3 and 4), which has several disadvantages. Readers might assume that each publication describes a unique patient cohort, and will be given the impression that the evidence-base for efficacy and safety of the intervention is much larger than it is in reality. Simultaneously, re-publication produces an exaggerated impression of the findings of these studies. Additionally, re-publication hampers evidence-synthesis, making it more difficult to perform and draw reliable conclusions from systematic reviews and meta-analyses. Of note, we have excluded all duplicate data from the efficacy results. We urge scientists to write one all-encompassing paper with all data after completion of a clinical trial. While we see merit in additional follow-up, authors should make this transparent and refer to previously published data, rather than re-publish the data. Mixing of cohorts from several studies may be feasible in some cases, but this should be clearly described and existing data should be preferably referenced, rather than re-published.

### Timing

We demonstrated that pre-clinical animal experiments were performed within the same timeframe as prospective clinical studies. This led to interesting findings such as that the single layer sphere (SLS) WEB device was implanted in humans well before animal experiments were published (Online Resources 3 and 4). It raises ethical concerns when clinical studies are actively carried out, while pre-clinical data are absent.

### Limitations

First, it was not possible to perform a meta-analysis as none of the included studies had a control group. This limits the extent of evidence synthesis. Second, although we have performed our search in two widely accepted databases for medical research, the use of additional databases might have led to the inclusion of additional papers. Third, we cannot rule out the existence of unpublished, industry-initiated, internal research documents assessing safety and efficacy of the WEB device. However, the *summary of safety and effectiveness data* report (https://www.accessdata.fda.gov/cdrh_docs/pdf17/P170032B.pdf),  which has led to FDA approval of the WEB device, suggests that the most important data has been included in our study. Besides a list of mandatory and unpublished in vivo and in vitro safety tests to adhere to ISO and ASTM standards regarding biocompatibility, 3 preclinical rabbit studies with a limited number of animals (*n* = 6, *n* = 8, *n* = 36) are briefly summarized. We suspect that one rabbit study (*n* = 36) has been included in our review, as the number of animals and the follow-up time points are the same, but we cannot be sure as it is not specified whether these animal studies have been published. One clinical study (WEB-IT), which is included in our study, has been used to demonstrate safety and efficacy in humans in the FDA report. We therefore think that most relevant data has been included in our study.

### Recommendations for future studies

- Use multiple animal models when testing (new) endovascular implants to assure that various aspects of the disease modeled are taken into account.
- Always report complications and deaths during pre-clinical studies and consider performing autopsies in animals at the end of a study to detect possible hidden safety issues.
- Use a negative control group to better estimate the effect of the device tested.
- Register your research protocol a priori in a publicly available database, such as preclinicaltrials.eu for animal studies and clinicaltrials.gov for clinical studies. Clearly define your study population, time points and outcome measures in your protocol.
- Use reporting guidelines when communicating your findings to the scientific community (ARRIVE for animal studies, STROBE for observational studies, etc.).
- Strive for clarity and comprehensiveness when publishing. When sharing results before the last follow-up moment, clearly state why it is important to publish now and refer to your study protocol to give guidance to readers what to expect from future studies. When reporting (additional) outcomes on a later time point, please clearly refer to the previously published results and cohort description.
- Only start with clinical studies when multiple animal studies have been completed to prevent inferior implants being tested in patients.

General recommendations such as using a power calculation to gauge the number of animals or patients needed to demonstrate an effect or the use of randomization and blinding throughout the study to avoid bias are always important and remain staples of ethical research practices.

## Conclusion

The rabbit elastase aneurysm model was the only pre-clinical animal model used to assess WEB device performance. Safety outcomes were not evaluated in animal studies and could therefore not be compared to clinical outcomes. Efficacy outcomes were more heterogeneous in animal studies than in clinical studies. Future research should focus on improving methodology and reporting in order to draw accurate conclusions on the performance of the WEB device.

## Supplementary Information

Below is the link to the electronic supplementary material.

Supplementary file1 (DOCX 13.7 KB)

Supplementary file2 (DOCX 17.6 KB)

Supplementary file3 (DOCX 37.9 KB)

Supplementary file4 (DOCX 21.8 KB)

## Abbreviations

WEB: Woven Endobridge
WNBA: Wide-neck bifurcation aneurysm
