# Supplementary material for: A systematic review of the Woven EndoBridge device—do findings in pre-clinical animal models compare to clinical results?
Source: Acta Neurochir (Wien). 2023 Jun 8;165(7):1869–79. doi: 10.1007/s00701-023-05638-y (PMC10319665; doi:10.1007/s00701-023-05638-y)
Supplement: Supplementary file 1 — Supplementary file1 (DOCX 13.7 KB) [file 701_2023_5638_MOESM1_ESM.docx]

**Online resource 1 – search strategy**

The search strategy used for this study.

| **Pubmed** | **07062021 392 hits** |
| --- | --- |
|  | ((((WEB[TiAb] OR device[tiab] OR devices[tiab] OR implant[tiab] OR implants[tiab]) AND (intrasaccular[TiAb] OR intra-saccular[tiab])) OR (WEB[TiAb] AND (aneurysm[mesh] OR aneurysm[TiAb] OR aneurysms[tiab]))) OR ("Woven EndoBridge" [TiAb] OR "Woven Endo-Bridge"[TiAb] OR "Woven EndoBridges" [TiAb] OR "Woven Endo-Bridges" [TiAb] OR "WEB device" [TiAb] OR "WEB devices" [TiAb] OR "WEB implant"[TiAb] OR "WEB implants"[TiAb])) NOT (review[ptyp]) |
|  |  |
| **EMBASE via Ovid** | **07062021 406 hits** |
|  | ((WEB OR device OR devices OR implant OR implants).ti,ab,kw AND (intrasaccular OR intra-saccular).ti,ab,kw) OR (WEB.ti,ab,kw AND ((exp aneurysm/) OR (aneurysm OR aneurysms).ti,ab,kw)) OR (("Woven EndoBridge" OR "Woven Endo-Bridge" OR "Woven EndoBridges" OR "Woven Endo-Bridges" OR "WEB device" OR "WEB devices" OR "WEB implant" OR "WEB implants").ti,ab,kw) NOT (review OR conference abstract).pt |

*Article title:*

A systematic review of the Woven EndoBridge device - do findings in pre-clinical animal models compare to clinical results?

*Journal name:*

Acta Neurochirurgica

*Author names:*

René Aquarius, PhD^1^

Danique Elbertsen

Joost de Vries, PhD

Hieronymus D. Boogaarts, PhD

Kimberley E. Wever, PhD

*Affiliation and e-mail address of the corresponding author:*

^1^Department of neurosurgery, Radboud University Medical Center, Nijmegen, Gelderland, The Netherlands. rene.aquarius@radboudumc.nl
