# Supplementary material for: A systematic review of the Woven EndoBridge device—do findings in pre-clinical animal models compare to clinical results?
Source: Acta Neurochir (Wien). 2023 Jun 8;165(7):1869–79. doi: 10.1007/s00701-023-05638-y (PMC10319665; doi:10.1007/s00701-023-05638-y)
Supplement: Supplementary file 2 — Supplementary file2 (DOCX 17.6 KB) [file 701_2023_5638_MOESM2_ESM.docx]

**Online resource 2 – reference list**

Reference list of all included studies.

1. Arthur AS, Molyneux A, Coon AL, Saatci I, Szikora I, Baltacioglu F, Sultan A, Hoit D, Delgado Almandoz JE, Elijovich L, Cekirge S, Byrne JV, Fiorella D, for the WEB-IT study investigators. The safety and effectiveness of the Woven EndoBridge (WEB) system for the treatment of wide-necked bifurcation aneurysms: final 12-month results of the pivotal WEB Intrasaccular Therapy (WEB-IT) Study. J Neurointerv Surg. 2019; 11: 924-930.
2. Bozzetto Ambrosi P, Gory B, Sivan-Hoffmann R, Riva R, Signorelli F, Labeyrie PE, Eldesouky I, Sadeh-Gonike U, Armoiry X, Turjman, F. Endovascular treatment of bifurcation intracranial aneurysms with the WEB SL/SLS: 6-month clinical and angiographic results. Interv Neuroradiol. 2015; 21: 462-469.
3. Cognard C, Januel, AC. Remnants and recurrences after the use of the WEB intrasaccular device in large-neck bifurcation aneurysms. Neurosurgery. 2015; 76: 522-530.
4. Ding Y, Dai D, Rouchaud A, Janot K, Asnafi S, Kallmes DF, Kadirvel R. WEB Device Shape Changes in Elastase-Induced Aneurysms in Rabbits. AJNR Am J Neuroradiol. 2021; 42: 334-339.
5. Ding YH, Dai D, Schroeder D, Kadirvel R, Kallmes DF. Experimental testing of the dual-layer Woven EndoBridge device using an elastase-induced aneurysm model in rabbits. Interv Neuroradiol. 2016; 22: 299-303.
6. Ding YH, Lewis DA, Kadirvel R, Dai D, Kallmes DF. The Woven EndoBridge: a new aneurysm occlusion device. AJNR Am J Neuroradiol. 2011; 32: 607-611.
7. Fiorella D, Molyneux A, Coon A, Szikora I, Saatci I, Baltacioglu F, Sultan A, Arthur A. Demographic, procedural and 30-day safety results from the WEB Intra-saccular Therapy Study (WEB-IT). J Neurointerv Surg. 2017; 9: 1191-1196.
8. Gherasim DN, Gory B, Sivan-Hoffmann R, Pierot L, Raoult H, Gauvrit JY, Desal H, Barreau X, Herbreteau D, Riva R, Ambesi Impiombato F, Armoiry X, Turjman F. Endovascular treatment of wide-neck anterior communicating artery aneurysms using WEB-DL and WEB-SL: short-term results in a multicenter study. AJNR Am J Neuroradiol. 2015; 36: 1150-1154.
9. Herbreteau D, Bibi R, Narata AP, Janot K, Papagiannaki C, Soize S, Pierot L. Are Anatomic Results Influenced by WEB Shape Modification? Analysis in a Prospective, Single-Center Series of 39 Patients with Aneurysms Treated with the WEB. AJNR Am J Neuroradiol. 2016; 37: 2280-2286.
10. Lubicz B, Mine B, Collignon L, Brisbois D, Duckwiler G, Strother C. WEB device for endovascular treatment of wide-neck bifurcation aneurysms. AJNR Am J Neuroradiol. 2013; 34: 1209-1214.
11. Pierot L, Costalat V, Moret J, Szikora I, Klisch J, Herbreteau D, Holtmannspötter M, Weber W, Januel AC, Liebig T, Sychra V, Strasilla C, Cognard C, Bonafé A, Molyneux A, Byrne JV, Spelle L. Safety and efficacy of aneurysm treatment with WEB: results of the WEBCAST study. J Neurosurg. 2016a; 124: 1250-1256.
12. Pierot L, Gubucz I, Buhk JH, Holtmannspötter M, Herbreteau D, Stockx L, Spelle L, Berkefeld J, Januel AC, Molyneux A, Byrne JV, Fiehler J, Szikora I, Barreau X. Safety and Efficacy of Aneurysm Treatment with the WEB: Results of the WEBCAST 2 Study. AJNR Am J Neuroradiol. 2017; 38: 1151-1155.
13. Pierot L, Moret J, Barreau X, Szikora I, Herbreteau D, Turjman F, Holtmannspötter M, Januel AC, Costalat V, Fiehler J, Klisch J, Gauvrit JY, Weber W, Desal H, Velasco S, Liebig T, Stockx L, Berkefeld J, Molyneux A, Byrne JV, Spelle L. Aneurysm Treatment With Woven EndoBridge in the Cumulative Population of 3 Prospective, Multicenter Series: 2-Year Follow-Up. Neurosurgery. 2020; 87: 357-367.
14. Pierot L, Moret J, Barreau X, Szikora I, Herbreteau D, Turjman F, Holtmannspötter M, Januel AC, Costalat V, Fiehler J, Klisch J, Gauvrit JY, Weber W, Desal H, Velasco S, Liebig T, Stockx L, Berkefeld J, Molyneux A, Byrne J, Spelle L. Safety and efficacy of aneurysm treatment with WEB in the cumulative population of three prospective, multicenter series. J Neurointerv Surg. 2018; 10: 553-559.
15. Pierot L, Moret J, Turjman F, Herbreteau D, Raoult H, Barreau X, Velasco S, Desal H, Januel AC, Courtheoux P, Gauvrit JY, Cognard C, Molyneux A, Byrne J, Spelle L. WEB Treatment of Intracranial Aneurysms: Clinical and Anatomic Results in the French Observatory. AJNR Am J Neuroradiol. 2016; 37: 655-659.
16. Pierot L, Moret J, Turjman F, Herbreteau D, Raoult H, Barreau X, Velasco S, Desal H, Januel AC, Courtheoux P, Gauvrit JY, Cognard C, Soize S, Molyneux A, Spelle L. WEB Treatment of Intracranial Aneurysms: Feasibility, Complications, and 1-Month Safety Results with the WEB DL and WEB SL/SLS in the French Observatory. AJNR Am J Neuroradiol. 2015; 36: 922-927.
17. Pierot, L, Spelle L, Molyneux A, Byrne J. Clinical and Anatomical Follow-up in Patients With Aneurysms Treated With the WEB Device: 1-Year Follow-up Report in the Cumulated Population of 2 Prospective, Multicenter Series (WEBCAST and French Observatory). Neurosurgery. 2016; 78: 133-141.
18. Pierot L, Szikora I, Barreau X, Holtmannspoetter M, Spelle L, Herbreteau D, Fiehler J, Costalat V, Klisch J, Januel AC, Weber W, Liebig T, Stockx L, Berkefeld J, Moret J, Molyneux A, Byrne J. Aneurysm treatment with WEB in the cumulative population of two prospective, multicenter series: 3-year follow-up. J Neurointerv Surg. 2021; 13: 363-368.
19. Rouchaud A, Brinjikji W, Ding YH, Dai D, Zhu YQ, Cloft HJ, Kallmes DF, Kadirvel R. Evaluation of the Angiographic Grading Scale in Aneurysms Treated with the WEB Device in 80 Rabbits: Correlation with Histologic Evaluation. AJNR Am J Neuroradiol. 2016; 37: 324-329.
20. Sivan-Hoffmann R, Gory B, Riva R, Labeyrie PE, Signorelli F, Eldesouky I, Gonike-Sadeh U, Armoiry X, Turjman F. One-Year Angiographic Follow-Up after WEB-SL Endovascular Treatment of Wide-Neck Bifurcation Intracranial Aneurysms. AJNR Am J Neuroradiol. 2015; 36: 2320-2324.
21. Struffert T, Lang S, Adamek E, Engelhorn T, Strother CM, Doerfler A. Angiographic C-arm CT visualization of the Woven EndoBridge cerebral aneurysm embolization device (WEB): first experience in an animal aneurysm model. Clin Neuroradiol. 2014; 24: 43-49.
22. Timsit C, Soize S, Benaissa A, Portefaix C, Gauvrit JY, Pierot L. Contrast-Enhanced and Time-of-Flight MRA at 3T Compared with DSA for the Follow-Up of Intracranial Aneurysms Treated with the WEB Device. AJNR Am J Neuroradiol. 2016; 37: 1684-1689.
23. Vardar Z, King RM, Kraitem A, Langan ET, Peterson LM, Duncan BH, Raskett CM, Anagnostakou V, Gounis MJ, Puri AS, Ughi GJ. High-resolution image-guided WEB aneurysm embolization by high-frequency optical coherence tomography. J Neurointerv Surg. 2020; 13: 669-673.

Article title:

A systematic review of the Woven EndoBridge device - do findings in pre-clinical animal models compare to clinical results?

Journal name:

Acta Neurochirurgica

Author names:

René Aquarius, PhD1

Danique Elbertsen

Joost de Vries, PhD

Hieronymus D. Boogaarts, PhD

Kimberley E. Wever, PhD

Affiliation and e-mail address of the corresponding author:

1Department of neurosurgery, Radboud University Medical Center, Nijmegen, Gelderland, The Netherlands. rene.aquarius@radboudumc.nl
