# Supplementary material for: A systematic review of the Woven EndoBridge device—do findings in pre-clinical animal models compare to clinical results?
Source: Acta Neurochir (Wien). 2023 Jun 8;165(7):1869–79. doi: 10.1007/s00701-023-05638-y (PMC10319665; doi:10.1007/s00701-023-05638-y)
Supplement: Supplementary file 3 — Supplementary file3 (DOCX 37.9 KB) [file 701_2023_5638_MOESM3_ESM.docx]

**Online resource 3 – characteristics of the included animal studies**

Characteristics of the included animal studies.

N/R: not reported. N/A: not applicable. M: male. WEB-SL: Woven EndoBridge Single Layer. WEB-DL: Woven EndoBridge Dual Layer. WEB-SLS: Woven EndoBridge Single Layer Sphere. CO: complete occlusion without angiographic visible collection of contrast within marker recess. MR: complete occlusion with angiographic visible collection of contrast within marker recess. NR: Neck remnant. AR: aneurysm remnant. NCO: near complete occlusion.

| **First author +**  **year of publication** | **Species** | **Strain** | **Number of animals used** | **Sex** | **Weight (kg)** | **Aneurysm induction method** | **Mean aneurysm dimensions (± SD) (mm)** | **Time between aneurysm induction and WEB placement** | **WEB types used** | **WEB sizes used** | **Angiographic occlusion outcome** | **Complications** |
| --- | --- | --- | --- | --- | --- | --- | --- | --- | --- | --- | --- | --- |
| Ding et al. 2021 | Rabbit | New Zealand White | 36 | N/R | N/R | Injection of elastase in the right common carotid artery | N/R | ≥ 3 weeks | WEB-DL (n= 12)  WEB-SL (n= 12) WEB-SLS (n= 12) | N/R | *3 months*  CO: n=5  MR: n=4  NR: n=3  AR: n=6  *12-months*  CO: n=5  MR: n=2  NR: n=3  AR: n=8 | N/R |
| Ding et al. 2016 | Rabbit | New Zealand White | 36 | N/R | N/R | Injection of elastase in the right common carotid artery | Neck: 3.4 (± 0.4)  Width: 3.9 (± 0.6) | ≥ 3 weeks | WEB-DL (n=36) | 5-10% larger than aneurysm width & length | *1 month*  CO: n=3  MR: n=3  NR: n=8  AR: n=4  *3 months*  CO: n=2  MR: n=2  NR: n=1  AR: n=1  *6 months*  CO: n=1  MR: n=2  NR: n=2  AR: n=1  *12 months*  CO: n=0  MR: n=3  NR: n=2  AR: n=1 | N/R |
| Ding et al. 2011 | Rabbit | New Zealand White | 24 | N/R | N/R | Injection of elastase in the right common carotid artery | N/R | ≥ 3 weeks | WEB-DL (n=24) | N/R | *1 month**  CO: n=2  NCO: n=3  AR: n=1  *3 months**  CO: n=3  NCO: n=2  AR: n=1  *6 months**  CO: n=1  NCO: n=5  AR: n=0  *12 months**  CO: n=2  NCO: n=4  AR: n=0  *data from individual timepoints requested and obtained from the authors | N/R |
| Rouchaud et al. 2016 | Rabbit | New Zealand White | 80 | N/R | N/R | Injection of elastase in the right common carotid artery | N/R | ≥ 3 weeks | WEB-DL (n= 36)  WEB-SL (n= 44) | N/R | *1 month**  CO: n=3  MR: n=1  NR: n=7  AR: n=15  *1.7 months**  CO: n=2  MR: n=0  NR: n=0  AR: n=3  *3 months**  CO: n=7  MR: n=12  NR: n=1  AR: n=10  *6 months**  CO: n=1  MR: n=2  NR: n=2  AR: n=7  *12 months**  CO: n=2  MR: n=2  NR: n=1  AR: n=2  *data from individual timepoints requested and obtained from the authors | N/A  Research focus on intra-observer and inter-observer variability regarding occlusion scores after WEB implantation. |
| Struffert et al. 2014 | Rabbit | New Zealand White | 5 | N/R | N/R | Injection of elastase in the right common carotid artery | N/R | 4 weeks | WEB-DL (n=5) | N/R | N/A  Research focus on visibility of WEB device using various CT protocols, not on efficacy or safety of the implant. | N/A  Research focus on visibility of WEB device using various CT protocols, not on efficacy or safety of the implant. |
| Vardar et al. 2020 | Rabbit | New Zealand White | 24 | M | 3.8 – 4.2 | Injection of elastase in the right common carotid artery | Neck: 2.7 (± 0.74)  Width: 3.5 (± 0.54) | ≥ 3 weeks | WEB-SL (n= 21) WEB-SLS (n=2) | SL W4-5-4 (n= 14) SL W4-6-5 (n= 7) SLS W4-6-S (n= 2) | *After implantation*  CO: n=0  MR: n=0  NR: n=0  AR: n=24  *1 month*  CO: n=2  MR: n=0  NR: n=4  AR: n=6  *2 months*  CO: n=1  MR: n=2  NR: n=4  AR: n=5  *3 months*  CO: n=3  MR: n=2  NR: n=8  AR: n=11 | N/R |

**References**

1. Ding Y, Dai D, Rouchaud A, Janot K, Asnafi S, Kallmes DF, Kadirvel R. WEB Device Shape Changes in Elastase-Induced Aneurysms in Rabbits. AJNR Am J Neuroradiol. 2021; 42: 334-339.
2. Ding YH, Dai D, Schroeder D, Kadirvel R, Kallmes DF. Experimental testing of the dual-layer Woven EndoBridge device using an elastase-induced aneurysm model in rabbits. Interv Neuroradiol. 2016; 22: 299-303.
3. Ding YH, Lewis DA, Kadirvel R, Dai D, Kallmes DF. The Woven EndoBridge: a new aneurysm occlusion device. AJNR Am J Neuroradiol. 2011; 32: 607-611.
4. Rouchaud A, Brinjikji W, Ding YH, Dai D, Zhu YQ, Cloft HJ, Kallmes DF, Kadirvel R. Evaluation of the Angiographic Grading Scale in Aneurysms Treated with the WEB Device in 80 Rabbits: Correlation with Histologic Evaluation. AJNR Am J Neuroradiol. 2016; 37: 324-329.
5. Struffert T, Lang S, Adamek E, Engelhorn T, Strother CM, Doerfler A. Angiographic C-arm CT visualization of the Woven EndoBridge cerebral aneurysm embolization device (WEB): first experience in an animal aneurysm model. Clin Neuroradiol. 2014; 24: 43-49.
6. Vardar Z, King RM, Kraitem A, Langan ET, Peterson LM, Duncan BH, Raskett CM, Anagnostakou V, Gounis MJ, Puri AS, Ughi GJ. High-resolution image-guided WEB aneurysm embolization by high-frequency optical coherence tomography. J Neurointerv Surg. 2020; 13: 669-673.

Article title:

A systematic review of the Woven EndoBridge device - do findings in pre-clinical animal models compare to clinical results?

Journal name:

Acta Neurochirurgica

Author names:

René Aquarius, PhD1

Danique Elbertsen

Joost de Vries, PhD

Hieronymus D. Boogaarts, PhD

Kimberley E. Wever, PhD

Affiliation and e-mail address of the corresponding author:

1Department of neurosurgery, Radboud University Medical Center, Nijmegen, Gelderland, The Netherlands. rene.aquarius@radboudumc.nl
