# Supplementary material for: A systematic review of the Woven EndoBridge device—do findings in pre-clinical animal models compare to clinical results?
Source: Acta Neurochir (Wien). 2023 Jun 8;165(7):1869–79. doi: 10.1007/s00701-023-05638-y (PMC10319665; doi:10.1007/s00701-023-05638-y)
Supplement: Supplementary file 4 — Supplementary file4 (DOCX 21.8 KB) [file 701_2023_5638_MOESM4_ESM.docx]

**Online resource 4 – characteristics of the included prospective clinical studies**

Characteristics of the included prospective clinical studies.

N/R: not reported. M: male. F: female. WEB-SL: Woven EndoBridge Single Layer. WEB-DL: Woven EndoBridge Dual Layer. WEB-SLS: Woven EndoBridge Single Layer Sphere. CO: complete occlusion without angiographic visible collection of contrast within marker recess. NR: Neck remnant. AR: aneurysm remnant. NCO: near complete occlusion. BA: basilar artery. ICA: internal carotid artery. MCA: middle cerebral artery. AcomA: anterior communicating artery. PcomA: posterior communicating artery. PA: pericallosal artery. VA-PICA: vertebral artery – posterior inferior cerebellar artery. VBA: vertebrobasilar artery. TIA: transient ischemic attack. DSA: digital subtraction angiography. 3D-TOF-MRA: 3 dimensional time-of-flight magnetic resonance angiography. CE-MRA: contrast enhanced magnetic resonance angiography.

Data presented in black are used to summarize data and create graphs.

| **First author +**  **year of publication** | **Number of patients included** | **Mean patient age (± SD) (years)** | **Sex** | **Aneurysm count per location** | **Mean aneurysm dimensions (± SD) (mm)** | **WEB types used (+ number of aneurysms)** | **WEB sizes used** | **Angiographic occlusion outcome** | **Complications** | **Remarks** |
| --- | --- | --- | --- | --- | --- | --- | --- | --- | --- | --- |
| Arthur et al. 2019 | 150 | 59 (± 10.2) | M: n=40  F: n=110 | BA tip: n=59  ICA tip: n=6  MCA bifurcation: n=45  AcomA: n=40 | Neck: 4.8 (± N/R)  Width: 6.4 (± N/R) | WEB-DL (n= 19)  WEB-SL (n= 107)  WEB-SLS (n= 22) | N/R | *12-months*  CO: n=77  NR: n=44  AR: n=22 | *≤ 30 days*  delayed ipsilateral parenchymal haemorrhage (n=1)  *> 30 days*  ischemic stroke (n=2)*  intracranial haemorrhage (n=1)  seizure (n=1)  TIA (n=3)  Recurrent TIAs (n=1)**  * 1 patient had 2 ischemic strokes at different time points due to pre-existing cerebrovascular disease  ** occurred after retreatment with pipeline embolization device | Same patient cohort as Fiorella et al. 2017.  Additional safety and efficacy data reported in this study due to longer follow-up. |
| Bozzetto et al. 2015 | 10 | 54.2 (± 8.9) | M: n=6  F: n=4 | BA tip: n=1  MCA bifurcation: n=4  AcomA: n=5 | Neck: 4.9 (± 1.2)  Width: 7.5 (± 1.9) | WEB-SL (n= 9)  WEB-SLS (n= 1) | 1 mm oversizing diameter + 1 mm undersizing length | *After implantation*  CO: n=2  NR: n=2  AR: n=6  *6 months*  CO: n=3  NR: n=6  AR: n=1 | *≤ 30 days*  None  *> 30 days*  None | Authors state in the methods section that angiographic follow-up should also take place at 3 months. However, these data are not presented. |
| Cognard et al. 2015 | 15 | 51.7 (± 6.6) | N/R | BA tip: n=2  ICA tip: n=1  MCA bifurcation: n=12 | Neck: 6.3 (± 1.5)  Width: 8.2 (± 2.4) | WEB-DL (n= 12)  WEB-SL (n= 3) | 1 mm oversizing diameter + 1 mm undersizing length | *After implantation*  CO: n=6  NR: n=8  AR: n=1  *3-6 months*  CO: n=1  NR: n=9  AR: n=4  *18-20 months*  CO: n=0  NR: n=4  AR: n=3 | N/R | All patients extracted from other patient cohorts: Pierot et al. 2015 (observatory) and Pierot et al. 2016a (WEBCAST).  None of the data from this inclusion used in any analyses. More complete overview given in Pierot et al. 2015 (observatory) and Pierot et al. 2016a (WEBCAST). |
| Fiorella et al. 2017 | 150 | 59 (± 10.2) | M: n=40  F: n=110 | BA tip: n=59  ICA tip: n=6  MCA bifurcation: n=45  AcomA: n=40 | Neck: 4.8 (± N/R)  Width: 6.4 (± N/R) | WEB-DL (n= 19)  WEB-SL (n= 107)  WEB-SLS (n= 22) | The most common device diameters implanted:  7 mm (n= 41)  8 mm (n= 31)  6 mm (n= 28)  5 mm (n= 28)  Sizing of 20 WEB devices N/R | N/A  Research focus on safety of the implant. | *≤ 30 days*  delayed ipsilateral parenchymal haemorrhage (n=1) | Same patient cohort as Arthur et al. 2019.  None of the data from this inclusion used in any analyses. More complete overview given in Arthur et al. 2019. |
| Gherasim et al. 2015 | 10 | 59.3 (± 8.7) | M: n=6  F: n=4 | AcomA: n=10 | Neck: 5.4 (± 1.3)  Width: 6.2 (± 1.2) | WEB-DL (n= 2)  WEB-SL (n= 5) | SL 7x3 (n=1)  SL 7x5 (n=2)  SL 7x6 (n=1)  SL 8x4 (n=1)  DL 7x4 (n=1)  DL 8x5 (n=1) | *After implantation*  CO: n=2  NR: n=4  AR: n=1  *3-6 months*  CO: n=3  NR: n=3  AR: n=1 | *≤ 30 days*  thromboembolism (n=1)*  * left A2 segment. Led to right-sided hemiparesis post-treatment, which resolved to a mild brachial deficit remnant at one month |  |
| Herbreteau et al. 2016 | 39 | 58.5 (± 20) | M: n=16  F: n=23 | BA tip: n=5  ICA tip: n=2  MCA bifurcation: n=21  AcomA: n=9  PcomA: n=1  PA: n=1 | Neck: 4.9 (± 1.3)  Width: 6.1 (± 1.3) | WEB-DL (n=16)  WEB-SL (n=18)  WEB-SLS (n=5) | undersized* WEB: n=11 patients  appropriately sized** WEB: n=28 patients  *undersized when the WEB diameter was not at least 0.5 mm greater than the aneurysm diameter.  ** appropriately sized when the WEB was at least 0.5 mm greater than the aneurysm diameter. | *After implantation*  CO: n=4  NR: n=13  AR: n=22  *3-6 months*  CO: n=23  NR: n=10  AR: n=5  *12 months*  CO: n=12  NR: n=8  AR: n=4  *≥18 months*  CO: n=13  NR: n=8  AR: n=3 | *≤ 30 days*  thromboembolic event (n=3)*  *> 30 days*  none  * resulting in permanent deficit (n=1) | Some patients (n=14, 36%) extracted from other patient cohorts: n=6 from Pierot et al. 2015 (observatory), n=5 from Pierot et al. 2016a (WEBCAST) and n=3 from Pierot et al 2017 (WEBCAST 2). |
| Lubicz et al. 2013 | 19 | 55.3 (± 9.6) | M: n=5  F: n=14 | BA tip: n=2  ICA tip: n=1  MCA bifurcation: n=14  AcomA: n=2  VA-PICA: n=1 | Neck: 6.5 (± 1.6)  Width: 8.1 (± 2.1) | N/R | the following statement was made:  “because of the lack of any experience with this type of device, we have chosen to be very conservative, trying not to oversize the device.” | *After implantation*  CO: n=1  NR: n=13  AR: n=5  *3 months*  CO: n=0  NR: n=13  AR: n=4  *6 months*  CO: n=0  NR: n=13  AR: n=1  *12 months*  CO: n=1  NR: n=4  AR: n=0 | *≤ 30 days*  gait instability* (n=1)  aneurysm rupture** (n=1)  *> 30 days*  none  * cause unknown, completely resolved at follow-up  ** resulted in a residual hemiparesis |  |
| Pierot et al. 2016a | 51 | 55.6 (± 10.8) | M: n=16  F: n=35 | BA tip: n=12  ICA tip: n=6  MCA bifurcation: n=29  AcomA: n=4 | Neck: 5.6 (± N/R)  Width: 8.2 (± N/R) | WEB-DL (n=48) | the following statement was made:  “appropriate device sizing was determined” | *After implantation*  CO: n=4  NR: n=12  AR: n=32  *6 months*  CO: n=23  NR: n=12  AR: n=6 | *≤ 30 days*  morbidity (mRS 3)* (n=1)  thromboembolic event** (n=1)  *> 30 days*  morbidity (mRS 1)* (n=1)  death*** (n=2)  * clinical status not described other than mRS and that it was related to the initial aneurysm rupture, according to authors  ** clinical status not described other than mRS (1) and that it was completely resolved at follow-up  *** both cases due to cancer | The original WEBCAST patient cohort. |
| Pierot et al. 2017 | 55 | 54.4 (± 10) | M: n=17  F: n=38 | BA tip: n=9  ICA tip: n=5  MCA bifurcation: n=25  AcomA: n=16 | Neck: 4.6 (± 1.1)  Width: 6.7 (± 2.3) | WEB-SL (n= 47)  WEB-SLS (n= 6) | N/R | *12 months*  CO: n=27  NR: n=13  AR: n=10 | *≤ 30 days*  intraoperative thromboembolic event* (n=8)  intraoperative aneurysm rupture** (n=1)  *> 30 days*  thromboembolic event*** (n=1)  death**** (n=1)  * resulting in no deficit (n=2), transient deficit (n=3) and permanent deficit (n=3)  ** asymptomatic  *** probably related to a history of stroke in that patient  **** retroperitoneal hematoma after the WEB procedure with additional complications | The original WEBCAST 2 patient cohort.  WEBCAST 2 pre-trial registration has the same identifier at clinicaltrials.gov (NCT01778322) as the WEBCAST study. |
| Pierot et al. 2018 | 168 | 55.5 (± 10.2) | M: n=56  F: n=112 | BA tip: n=30  ICA tip: n=17  MCA bifurcation: n=86  AcomA: n=36 | Neck: 5.2 (± 1.6)  Width: 7.6 (± 2.5) | WEB-DL (n=78)  WEB-SL & WEB-SLS combined (n=85) | the following statement was made:  “Appropriate device sizing was determined based on 2D and 3D digital subtraction angiography (DSA).” | *12 months*  CO: n=81  NR: n=40  AR: n=32 | *≤ 30 days*  thromboembolic event* (n=24)  intraoperative aneurysm rupture (n=2)  intracranial haemorrhage (n=1)  *> 30 days*  death** (n=5)  thromboembolic event*** (n=1)  * resulting in no deficit (n=11), transient deficit (n=8) and permanent deficit (n=5)  ** cause of death: cancer (n=2), cirrhosis (n=1), progressive worsening of mass effect (n=1), retroperitoneal hematoma after the WEB procedure with additional complications (n=1)  *** this event occurred during retreatment with a flow diverter | All patients extracted from other patient cohorts: Pierot et al. 2015 (observatory), Pierot et al. 2016a (WEBCAST) and Pierot et al. 2017 (WEBCAST 2).  Clinical follow-up at 1 month was already published for all 3 cohorts.  12 month radiological and safety follow-up already published for the WEBCAST 2 cohort.  Additional safety and efficacy data reported in this study due to longer follow-up for some studies at the time of publishing. |
| Pierot et al. 2020 | 168 | 55.5 (± 10.2) | M: n=56  F: n=112 | BA tip: n=30  ICA tip: n=17  MCA bifurcation: n=86  AcomA: n=36 | Neck: 5.2 (± 1.6)  Width: 7.6 (± 2.5) | WEB-DL (n=78)  WEB-SL & WEB-SLS combined (n=85) | the following statement was made:  “Selection of the device size was made using 2-dimensional and 3-dimensional digital subtraction angiography (DSA).” | *24 months*  CO: n=62  NR: n=36  AR: n=23 | *≤ 30 days*  all reported in Pierot et al. 2018  *> 30 days*  up to 1 year reported in Pierot et al. 2018.  between 1-2 years: none | All patients extracted from other patient cohorts: Pierot et al. 2015 (observatory), Pierot et al. 2016a (WEBCAST) and Pierot et al. 2017 (WEBCAST 2).  Additional safety and efficacy data reported in this study due to longer follow-up. |
| Pierot et al. 2016b | 62 | 56.6 (± 9.8) | M: n=23  F: n=39 | BA tip: n=9  ICA tip: n=6  MCA bifurcation: n=32  AcomA: n=16 | Neck: ≥4mm (n=57 aneurysms)  Wirdth: <10mm (n=52 aneurysms) | WEB-DL (n=31)  WEB-SL & WEB-SLS combined (n=32) | the following statement was made:  “Appropriate device sizing was selected on the basis of 2D and 3D digital subtraction angiography” | *12 months*  CO: n=30  NR: n=16  AR: n=12 | *≤ 30 days*  thromboembolic event* (n=9)  worsening of pre-existing mass effect (n=1)  intraoperative rupture** (n=1)  intracranial haemorrhage*** (n=1)  *> 30 days*  death**** (n=2)  * resulting in permanent deficit (n=1)  ** asymptomatic  *** unrelated  **** cause of death: progressive worsening of mass effect (n=1), unrelated (n=1) | Same patient cohort as Pierot et al. 2015.  Additional safety and efficacy data reported in this study due to longer follow-up.  Number of thromboembolic events reported here does not match those reported in Pierot et al. 2015. |
| Pierot et al. 2015 | 62 | 56.6 (± 9.8) | M: n=23  F: n=39 | BA tip: n=9  ICA tip: n=6  MCA bifurcation: n=32  AcomA: n=16 | Neck: ≥4mm (n=57 aneurysms)  Wirdth: <10mm (n=52 aneurysms) | WEB-DL (n=31)  WEB-SL & WEB-SLS combined (n=32) | the following statement was made:  “After accurate evaluation of aneurysm anatomy […], it was determined whether the treatment with the WEB was indicated and device sizing was appropriate.” | N/A  Safety study | *≤ 30 days*  thromboembolic event* (n=10)  worsening of pre-existing mass effect (n=1)  intraoperative rupture** (n=1)  intracranial haemorrhage*** (n=1)  * resulting in no deficit (n=4), transient deficit (n=5) and permanent deficit (n=1)  ** asymptomatic  *** unrelated and asymptomatic | The original French observatory patient cohort.  Same patient cohort as Pierot et al. 2016b.  Number of thromboembolic events reported here do not match those reported in Pierot et al. 2016b. |
| Pierot et al. 2016c | 113 | 56.1 (± 10.2) | M: n=39  F: n=74 | BA tip: n=21  ICA tip: n=12  MCA bifurcation: n=21  AcomA: n=60 | Neck: 5.6 (± 0.44)  Width: 8.0 (± 2.4) | WEB-DL (n= 82)  WEB-SL & WEB-SLS combined (n= 32) | the following statement was made:  “Appropriate device sizing was determined based on 2- and 3-dimensional digital subtraction angiography (DSA).” | *After implantation*  CO: n=15  NR: n=20  AR: n=79  *6 months*  CO: n=23  NR: n=12  AR: n=6  *12 months*  CO: n=56  NR: n=26  AR: n=18 | *≤ 30 days*  thromboembolic event* (n=17)  worsening of pre-existing mass effect (n=1)  intraoperative rupture** (n=1)  intracranial haemorrhage*** (n=1)  *> 30 days*  death**** (n=4)  * resulting in no deficit (n=4), transient deficit (n=5) and permanent deficit (n=1)  ** asymptomatic  *** unrelated and asymptomatic  **** cause of death: cancer (n=2), cirrhosis (n=1), progressive worsening of mass effect (n=1). | All patients extracted from other patient cohorts: Pierot et al. 2015 (observatory) amd Pierot et al. 2016a (WEBCAST).  6 month occlusion data only available for WEBCAST cohort. These numbers do not match the numbers published in Pierot et al. 2016a.  12 month occlusion data for observatory already published in Pierot et al. 2016b and partially published for WEBCAST cohort in Herbreteau et al. 2016. |
| Pierot et al. 2021 | 106 | 56 (± 10.4) | M: n=33  F: n=73 | BA tip: n=21  ICA tip: n=11  MCA bifurcation: n=54  AcomA: n=20 | Neck: 5.1 (± ?)*  Width: 7.4 (± ?)*  * SD unknown, not reported in Pierot et al. 2016a | WEB-DL (n=48)  WEB-SL (n= 47)  WEB-SLS (n= 6) | in Pierot et al. 2016a the following statement was made:  “appropriate device sizing was determined”  no statement regarding device sizing was made in Pierot et al. 2017 | *36 months*  CO: n=31  NR: n=20  AR: n=10 | *≤ 30 days*  all reported in Pierot et al. 2018  *> 30 days*  up to 2 year reported in Pierot et al. 2018 and Pierot et al. 2020.  no delayed complications, hemorrhagic or thromboembolic events reported during the 2–3 year period following the initial procedure  death at 3 years* (n=5)  * not different from previously reported deaths in Pierot et al. 2018: procedure- related retroperitoneal hematoma (n=1), cancer (n=3), pneumonia (n=1) | All patients extracted from other patient cohorts: Pierot et al. 2016a (WEBCAST) and Pierot et al. 2017 (WEBCAST 2).  Additional safety and efficacy data reported in this study due to longer follow-up. |
| Sivan et al. 2015 | 8 | 56.1 (± 8) | M: n=5  F: n=3 | BA tip: n=1  MCA bifurcation: n=3  AcomA: n=4 | Neck: 4.9 (± 1.2)  Width: 6.3 (± 1.2) | WEB-SL (n= 7)  WEB-SLS (n= 1) | SL 6x3 (n=1)  SL 6x4 (n=1)  SL 7x5 (n=2)  SL 7x6 (n=1)  SL 8x4 (n=1)  SL 9x5 (n=1)  SLS 7x4 (n=1) | *6 months*  CO: n=1  NR: n=6  AR: n=1  *12 months*  CO: n=1  NR: n=6  AR: n=1 | *≤ 30 days*  none  *> 30 days*  none |  |
| Timsit et al. 2016 | 26 | 55 (± 10) | M: n=4  F: n=22 | ICA tip: n=6  MCA bifurcation: n=7  AcomA: n=9  VBA: n=4 | size: ≤ 5 mm (n= 1 aneurysm)  size:5 - 10 mm (n= 22 aneurysms)  size: ≥ 10 mm (n= 3 aneurysms) | WEB-DL (n= 14)  WEB-SL (n= 8)  WEB-SLS (n= 4) | N/R | *6-15 months*  DSA  CO: n=12  NR: n=10  AR: n=4  3D-TOF-MRA  CO: n=17  NR: n=8  AR: n=1  CE-MRA  CO: n=18  NR: n=7  AR: n=1 | N/A  research focus on inter-observer variability for the assessment of aneurysm occlusion. | Strong suspicion that (some of) these patients were already included in the French observatory, WEBCAST and WEBCAST 2 cohorts, but this is not mentioned in the study.  Patients were included from June 2011 to December 2014 in the University Hospital in Reims. At the same center and during the same period, patients were included in the French observatory, WEBCAST and WEBCAST 2 cohorts. |

**References**

1. Arthur AS, Molyneux A, Coon AL, Saatci I, Szikora I, Baltacioglu F, Sultan A, Hoit D, Delgado Almandoz JE, Elijovich L, Cekirge S, Byrne JV, Fiorella D, for the WEB-IT study investigators. The safety and effectiveness of the Woven EndoBridge (WEB) system for the treatment of wide-necked bifurcation aneurysms: final 12-month results of the pivotal WEB Intrasaccular Therapy (WEB-IT) Study. J Neurointerv Surg. 2019; 11: 924-930.
2. Bozzetto Ambrosi P, Gory B, Sivan-Hoffmann R, Riva R, Signorelli F, Labeyrie PE, Eldesouky I, Sadeh-Gonike U, Armoiry X, Turjman, F. Endovascular treatment of bifurcation intracranial aneurysms with the WEB SL/SLS: 6-month clinical and angiographic results. Interv Neuroradiol. 2015; 21: 462-469.
3. Cognard C, Januel, AC. Remnants and recurrences after the use of the WEB intrasaccular device in large-neck bifurcation aneurysms. Neurosurgery. 2015; 76: 522-530.
4. Fiorella D, Molyneux A, Coon A, Szikora I, Saatci I, Baltacioglu F, Sultan A, Arthur A. Demographic, procedural and 30-day safety results from the WEB Intra-saccular Therapy Study (WEB-IT). J Neurointerv Surg. 2017; 9: 1191-1196.
5. Gherasim DN, Gory B, Sivan-Hoffmann R, Pierot L, Raoult H, Gauvrit JY, Desal H, Barreau X, Herbreteau D, Riva R, Ambesi Impiombato F, Armoiry X, Turjman F. Endovascular treatment of wide-neck anterior communicating artery aneurysms using WEB-DL and WEB-SL: short-term results in a multicenter study. AJNR Am J Neuroradiol. 2015; 36: 1150-1154.
6. Herbreteau D, Bibi R, Narata AP, Janot K, Papagiannaki C, Soize S, Pierot L. Are Anatomic Results Influenced by WEB Shape Modification? Analysis in a Prospective, Single-Center Series of 39 Patients with Aneurysms Treated with the WEB. AJNR Am J Neuroradiol. 2016; 37: 2280-2286.
7. Lubicz B, Mine B, Collignon L, Brisbois D, Duckwiler G, Strother C. WEB device for endovascular treatment of wide-neck bifurcation aneurysms. AJNR Am J Neuroradiol. 2013; 34: 1209-1214.
8. Pierot L, Costalat V, Moret J, Szikora I, Klisch J, Herbreteau D, Holtmannspötter M, Weber W, Januel AC, Liebig T, Sychra V, Strasilla C, Cognard C, Bonafé A, Molyneux A, Byrne JV, Spelle L. Safety and efficacy of aneurysm treatment with WEB: results of the WEBCAST study. J Neurosurg. 2016a; 124: 1250-1256.
9. Pierot L, Gubucz I, Buhk JH, Holtmannspötter M, Herbreteau D, Stockx L, Spelle L, Berkefeld J, Januel AC, Molyneux A, Byrne JV, Fiehler J, Szikora I, Barreau X. Safety and Efficacy of Aneurysm Treatment with the WEB: Results of the WEBCAST 2 Study. AJNR Am J Neuroradiol. 2017; 38: 1151-1155.
10. Pierot L, Moret J, Barreau X, Szikora I, Herbreteau D, Turjman F, Holtmannspötter M, Januel AC, Costalat V, Fiehler J, Klisch J, Gauvrit JY, Weber W, Desal H, Velasco S, Liebig T, Stockx L, Berkefeld J, Molyneux A, Byrne JV, Spelle L. Aneurysm Treatment With Woven EndoBridge in the Cumulative Population of 3 Prospective, Multicenter Series: 2-Year Follow-Up. Neurosurgery. 2020; 87: 357-367.
11. Pierot L, Moret J, Barreau X, Szikora I, Herbreteau D, Turjman F, Holtmannspötter M, Januel AC, Costalat V, Fiehler J, Klisch J, Gauvrit JY, Weber W, Desal H, Velasco S, Liebig T, Stockx L, Berkefeld J, Molyneux A, Byrne J, Spelle L. Safety and efficacy of aneurysm treatment with WEB in the cumulative population of three prospective, multicenter series. J Neurointerv Surg. 2018; 10: 553-559.
12. Pierot L, Moret J, Turjman F, Herbreteau D, Raoult H, Barreau X, Velasco S, Desal H, Januel AC, Courtheoux P, Gauvrit JY, Cognard C, Molyneux A, Byrne J, Spelle L. WEB Treatment of Intracranial Aneurysms: Clinical and Anatomic Results in the French Observatory. AJNR Am J Neuroradiol. 2016; 37: 655-659.
13. Pierot L, Moret J, Turjman F, Herbreteau D, Raoult H, Barreau X, Velasco S, Desal H, Januel AC, Courtheoux P, Gauvrit JY, Cognard C, Soize S, Molyneux A, Spelle L. WEB Treatment of Intracranial Aneurysms: Feasibility, Complications, and 1-Month Safety Results with the WEB DL and WEB SL/SLS in the French Observatory. AJNR Am J Neuroradiol. 2015; 36: 922-927.
14. Pierot, L, Spelle L, Molyneux A, Byrne J. Clinical and Anatomical Follow-up in Patients With Aneurysms Treated With the WEB Device: 1-Year Follow-up Report in the Cumulated Population of 2 Prospective, Multicenter Series (WEBCAST and French Observatory). Neurosurgery. 2016; 78: 133-141.
15. Pierot L, Szikora I, Barreau X, Holtmannspoetter M, Spelle L, Herbreteau D, Fiehler J, Costalat V, Klisch J, Januel AC, Weber W, Liebig T, Stockx L, Berkefeld J, Moret J, Molyneux A, Byrne J. Aneurysm treatment with WEB in the cumulative population of two prospective, multicenter series: 3-year follow-up. J Neurointerv Surg. 2021; 13: 363-368.
16. Sivan-Hoffmann R, Gory B, Riva R, Labeyrie PE, Signorelli F, Eldesouky I, Gonike-Sadeh U, Armoiry X, Turjman F. One-Year Angiographic Follow-Up after WEB-SL Endovascular Treatment of Wide-Neck Bifurcation Intracranial Aneurysms. AJNR Am J Neuroradiol. 2015; 36: 2320-2324.
17. Timsit C, Soize S, Benaissa A, Portefaix C, Gauvrit JY, Pierot L. Contrast-Enhanced and Time-of-Flight MRA at 3T Compared with DSA for the Follow-Up of Intracranial Aneurysms Treated with the WEB Device. AJNR Am J Neuroradiol. 2016; 37: 1684-1689.

Article title:

A systematic review of the Woven EndoBridge device - do findings in pre-clinical animal models compare to clinical results?

Journal name:

Acta Neurochirurgica

Author names:

René Aquarius, PhD1

Danique Elbertsen

Joost de Vries, PhD

Hieronymus D. Boogaarts, PhD

Kimberley E. Wever, PhD

Affiliation and e-mail address of the corresponding author:

1Department of neurosurgery, Radboud University Medical Center, Nijmegen, Gelderland, The Netherlands. rene.aquarius@radboudumc.nl
